# A Review Article: The Relationship Between Obesity and Colorectal Cancer

**DOI:** 10.1007/s11892-024-01556-0

**Published:** 2024-12-02

**Authors:** Lily Nguyen, Skandan Shanmugan

**Affiliations:** Department of Surgery, Division of Colon and Rectal Surgery, University of California, 333 The City Blvd West, Suite 1600, Suite 1600, Irvine, CA USA 92868-3298

**Keywords:** Obesity, Colorectal cancer, Bariatric surgery, Prehabilitation, Rectal cancer, Colorectal cancer surgery

## Abstract

**Purpose of Review:**

This article aims to review the recent literature assessing the relationship between obesity and colorectal carcinogenesis, the effect of obesity on the treatment of colorectal cancer (CRC), tools available to help augment the increased risk, and outcomes for patients who are affected by both obesity and colorectal cancer.

**Recent Findings:**

The biochemical mechanisms contributing to CRC carcinogenesis are not well understood but are suspected to be related to adipose tissue leading to a pro-inflammatory state and changes in the gut microbiome. Individuals with obesity are at higher risk for CRC development, worse oncologic outcomes, and increased rates of post-operative complications. Bariatric surgery decreases CRC risk but results with GLP-1 agonists are heterogeneous. Prehabilitation is the only weight loss method that has been demonstrated to decrease risks of post-operative morbidity in this population.

**Summary:**

Obesity augments CRC risk and outcomes. There are persistent knowledge gaps in etiology and epidemiology for the increased CRC risk in obese patients and more research is required to identify the therapeutic advantage of weight loss on CRC risk.

## Introduction

According to the World Health Organization (WHO), over 2.5 billion adults were overweight and 890 million had obesity in 2022 [1]. The National Institute of Health (NIH) and WHO use Body Mass Index (BMI) as a statistical index assessing an individual’s weight and height to estimate body fat. BMI is then further characterized into four categories – underweight, normal weight, overweight, or obese [2]. In White, Black, and Hispanic individuals, overweight is defined as BMI greater than or equal to 25 to 29.9 kg/m^2^. Obesity –further classified into class I to III – is characterized as a BMI greater than or equal to 30 kg/m^2 ^[2].

Despite national and international health initiatives, the rate of obesity has increased over the past several decades [3]. Obesity is associated with many metabolic and cardiopulmonary disorders including cardiovascular disease, type 2 diabetes (T2D), hypertension, dyslipidemia, metabolic dysfunction-associated steatotic liver disease (MASLD) and obstructive sleep apnea [4]. Obesity is a known risk factor for the development of colorectal cancer, and many other malignancies including breast, endometrial, esophageal, gallbladder, gastric, kidney, bladder, liver, ovarian, pancreas, thyroid, prostate, oropharyngeal cancers in addition to meningiomas and multiple myeloma [5]. Not only has obesity been linked to the development of both solid organ and hematologic malignancies, but it may also associated with increased risk of mortality overall in patients with cancer – excluding lung cancer, renal cell cancer, and melanoma [6]. Therefore, management of obesity is critical in both the prevention and treatment of many, if not all, cancers.

Colorectal cancer (CRC) is the third most diagnosed cancer worldwide and is the second leading cause of cancer-related deaths [7]. There is ongoing research assessing modifiable lifestyle factors like obesity and its role in colorectal carcinogenesis and cancer outcomes as both the global cancer burden and the rates of obesity continually increase [3]. Childhood and early adulthood obesity has been linked to early-onset colorectal cancer [8–10]. Additionally, there is increasing research on visceral adipose tissue (VAT) contributing to CRC risk due to systemic low-grade inflammation and direct interaction with the tumor microenvironment [11]. Furthermore, obesity has been linked to worse overall CRC outcomes, increasing morbidity and mortality with surgical intervention [6, 12, 13].

This article aims to review the recent literature assessing the relationship between obesity and colorectal carcinogenesis, the effect of obesity on the treatment of colorectal cancer, tools available to help augment the increased risk, and outcomes for patients who are affected by both obesity and colorectal cancer.

## Methods

### Search Strategy and Inclusion Criteria

The terms “obesity + colorectal cancer OR obesity + colon cancer OR obesity + rectal cancer” were searched in PubMed. The review of the literature included full-text articles that were published in English between September 2019 to September 2024. A total of 1,747 results were found. Additionally, sources cited within the identified literature reviews were also sourced.

## Epidemiology of Obesity and Colorectal Cancer

Most recent studies utilize BMI to characterize obesity when assessing the relationship with CRC [5, 8, 14]. Body size traits such as waist circumference, visceral adipose tissue, and body fat percentage have also been linked to CRC risk [15]. According to data from the 2017–2018 National Health and Nutrition Examination Survey (NHANES), nearly 1 in 3 adults are overweight (30.7%), over 2 in 5 adults have obesity (42.4%), and approximately 1 in 11 adults (9.2%) experience severe obesity. Adult men are more likely to be overweight compared to women (34.1% vs. 27.5%), while adult women have a higher prevalence of severe obesity (11.5% vs. 6.9%) [16]. Nearly 1 in 2 Black and Hispanic adults are affected by obesity compared to 2 in 5 non-Hispanic White adults [16].

**Fig. 1:**
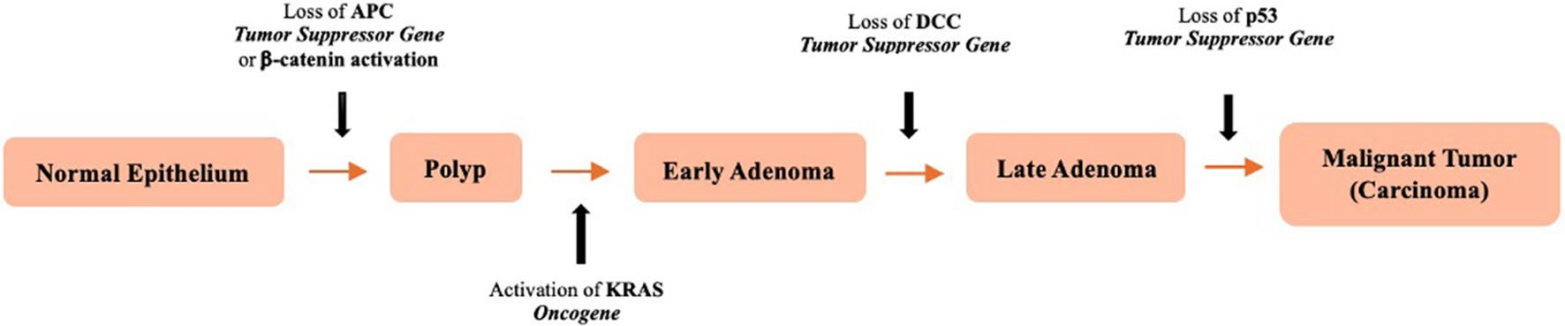
Adenoma-carcinoma sequence for sporadic colorectal tumorigenesis

According to WHO, CRC was the third most common cancer worldwide (1.93 million cases) and second most common cause of cancer-related death worldwide (916,000 deaths) in 2020 [17]. CRC age-standardized incidence rates are observed at a higher incidence in economically more developed regions, traditionally “westernized” countries, including Australia/New Zealand (women 32.2 and men 44.8 per 100,000), Europe (women 32.6 and men 37.3), and Northern America (women 22.7 and men 30.1) [18]. Only approximately 5–10% of CRC cases are associated with inherited causes such as familial adenomatous polyposis and hereditary non-polyposis [18, 19]. The majority of CRC cases are sporadic and are attributed to genetic and environmental causes such as diets high in red and processed meats, alcohol consumption, and obesity including abdominal and general obesity [9, 10, 18]. The underlying pathogenesis of sporadic CRC tumorigenesis and progression is the adenoma-carcinoma sequence. It begins with changes in the epithelium caused by genetic mutations in oncogenes (i.e. K-ras) and tumor suppressor genes (i.e. APC or BRAF) to form adenomas. If this process progresses, then adenomas can become dysplastic and eventually progress to carcinoma [20] (Fig. 1).

Historically, the first study that quantitatively assessed the relationship between obesity and risk of CRC development was in 2000 by Bergstrom et al. Authors found that excess body weight increased the risk of cancer for endometrial, kidney, breast, gallbladder, and colon cancer with the largest number of attributable cases for colon cancer. They also found relative risk (RR) of 1.03 per unit increase in BMI. Of note, these studies assessed primarily “westernized” countries including USA, Canada, UK, Sweden, and Australia [21, 22]. Research on this topic has expanded significantly in the interim, with many epidemiologic meta-analyses investigating the association between general obesity and visceral obesity and risk of CRC development using BMI, VAT, body fat percentage, and waist-to-hip ratio (WHR) in the past 5 years Table 1.

**Table 1:** Summary of studies investigating the relationship between general obesity and VAT with CRC development from 2019–2024

Study	Study Design	Number of Studies	Total Sample Size (*n*)^	Obesity Metric*	Relative Risk	CI (95%)	Key Findings
Lei et al. (2020)	Meta-Analysis	15	520,091	BMI	1.32	1.11–1.56	Obesity at a young age led to a 32% increased risk of CRC, each 1 kg/m^2^ increment was associated with a 2% increased risk, although this effect may not be as strong in women
O’Sullivan et al. (2022) [33]	Meta-Analysis	20	42,692	BMI	1.54	1.01–2.35	Obesity was significantly associated with development of CRC, but with marked heterogeneity among risk estimates
Garcia and Song (2019) [35]	Meta-analysis	15	15,288	BMI	1.39 (male) 1.19 (female)	1.20–1.62 1.06–1.35	Early-life obesity increased risk of CRC in adult, with a stronger association found in males
Li et al. (2021) [25]	Meta-Analysis	6	242,561	BMI	1.88	1.40–2.54	Obesity was strongly associated with increased risk of CRC
Li et al. (2022) [8]	Case–Control	–	6,602	BMI	3.29 (20 years old) 2.15 (30 years old) 1.75 (10 years prior to diagnosis)	1.08–10.09 1.16–4.01 1.02–2.99	A dose–response relationship exists with BMI at different ages with early-onset CRC risk
Bardou et al. (2022) [23]	Review Article	–	–	BMI	–	–	Any 1 kg/m^2^ increase in BMI confers more risk in CRC development (HR 1.03)
Hua et al. (2023) [24]	Meta-Analysis	30	66,312	BMI VAF	1.52 1.22	1.20–1.91 1.16–1.30	Both obesity and abdominal obesity demonstrated a positive associated with CRC
Chaplin et al. (2022) [11]	Review Article	–	–	VAT	–	–	VAT directly interacts with the tumor microenvironment (TME) and can lead to a tumorigenic TME due to multiple factors contributing to an inflammatory state
Mandic et al. (2023) [27]	Review Article of Systemic Reviews and Meta-Analyses	18	–	BMI	–	–	Pre-diagnostic cancer-related weight loss ^^ may be a potential source of bias leading to underestimation of the risk of obesity and CRC
Rontogianni et al. (2024) [28]	Mendelian Randomization	–	98,715	WHI	1.20	1.03–1.39	Genetically predicted WHI had a positive association with CRC and was similar between subsites (colon and rectum) and sex

*CI* confidence interval, *^n* number of patients included, *BMI body mass index, *VAT* visceral adipose tissue, *VAF* visceral abdominal fat, *WHI* waist-to-hip index; –not applicable,; ^^ = up to two years prior to diagnosis or interview, with association decreasing from earlier time frames

In summary, there is strong evidence supporting the positive risk association between obesity and CRC [8, 11, 23–25]. Many studies also demonstrate a positive risk association with other modifiable lifestyle factors such as alcohol use, smoking, consumption of processed foods, and a low-fiber diet [23, 26–28]. Obesity prevalence has been increasing nationally, with a greater increase observed in women > men and in Non-Hispanic Black > Non-Hispanic Whites [16, 26]. Obesity is also more prevalent when comparing Hispanic Americans to Non-Hispanic White Americans at a ratio of 1.2 in 2018 [29]. Racial and ethnic disparities have been observed when assessing CRC incidence and mortality [30]. Based on SEER data, for every 100 cases of CRC in White Americans, there are 113 cases of CRC in Black Americans. Furthermore, the mortality ratio for Black Americans is 1.32 compared to White Americans with the incidence curve favoring earlier ages demonstrating significant disparity in this population [30]. Between 1993–2007, there was a greater increase in incidence of CRC observed in younger Hispanics (< 50 years old) compared to non-Hispanic whites (45% vs. 27%). Proposed underlying factors may include low adherence to CRC screening, absence of health insurance, and lower socioeconomic status [31].

Between 1988–1994 and 2017–2018, the prevalence of obesity amongst children and adolescents aged 2 to 19 years old roughly doubled [16]. There are multiple studies linking early-onset colorectal cancer (EoCRC) with obesity at younger ages [32–34]. Lei et al. found a 32% increased risk of CRC (95% CI: 1.11–1.56) in young adults with obesity, however, the correlation was not observed in women (RR: 1.22, 95% CI 0.99–1.51). Nevertheless, dose–response analysis showed that for every 1 kg/m^2^ increment of BMI, there was a 2% overall risk of CRC development [32]. A different systematic review by O’Sullivan et al. found a 54% increased risk of EoCRC with obesity (RR 1.54, 95% CI 1.01–2.35). They also found that demographic factors such as Caucasian ethnicity (1.31 RR, 95% CI 1.06–1.62), male sex (1.59 RR, 95% CI 1.23–2.07), and a history of CRC in a first-degree relative (4.21 RR, 95% CI 2.61–6.79) were significantly associated with CRC development [33]. This suggests that weight control is crucial for young individuals in augmenting their risk for CRC and reaffirmed that demographic factors play a significant role in the carcinogenesis of CRC.

Early-onset obesity appears to affect males and females at different magnitudes. Garcia and Song (2019) performed a meta-analysis that showed a 39% increased risk (95% CI 1.20–1.62) for adult CRC development in males with early-onset obesity and a 19% increased risk (95% CI 1.06–1.35) in females. Males with early-onset obesity more frequently developed cancers of the distal colon (1.51 RR, 95% CI 1.22–1.97) and the rectum (1.39 RR, 95% 1.10–1.77). In females, early-onset obesity was more strongly associated with development of rectal cancer (1.38 RR, 95% CI 0.94–2.03) [35]. However, limitations of this article included many studies that asked participants to recall their childhood BMI which is subject to recall bias. Nevertheless, this is overall consistent with the fact that male sex is a significant independent positive risk factor for CRC [33].

## Obesity and the Tumor Microenvironment

The underlying mechanisms linking obesity and colorectal carcinogenesis are complex and not yet fully understood. It is believed that adipose tissue leads to a generalized inflammatory state in obesity and may be the key to understanding this relationship. VAT has been associated with metabolic changes such as insulin resistance, hyperglycemia, oxidative stress, and dysregulation of adipokines like adiponectin and leptin [11].

Adiponectin, an insulin-sensitizing hormone, has an inverse relationship with visceral obesity. Current research suggests that low adiponectin level is linked with poor CRC outcome and studies that suggest a possible correlation between higher levels of adiponectin and improved overall outcome in CRC [11, 36, 37]. In vitro*,* adiponectin has demonstrated an anti-proliferative effect on colon cancer cells [11]. However, observational studies have also reported increased systemic adiponectin levels in individuals with CRC [36]. Thus, currently it is not clearly known whether a causal or protective relationship exists between adiponectin and CRC.

Leptin is produced by adipose tissue and is sometimes considered the “anti-obesity” hormone due to its role in regulating energy balance and suppressing appetite [38]. It is an insulin-sensitizing hormone and acts as an inflammatory mediator leading to a proinflammatory cytokine production in macrophages [11]. Leptin’s receptors under the group *LEPR* has also been linked to CRC proliferation. Lack of LEPR expression has been demonstrated to decrease tumor proliferation in CRC, while leptin itself has both a mitogenic and anti-apoptotic effect leading to increased tumorigenesis [38]. Leptin has also found to be abnormally expressed in the setting of obesity, leading to increased leptin concentrations, leptin resistance, and impaired binding to leptin receptor [39]. Nevertheless, the relationship between leptin, obesity, and CRC is not fully understood. Itaconate, a mitochondrial metabolite, affects cellular pathways that are also regulated by leptin. Itaconate contributes to downregulation of peroxisome proliferator-activated receptor-gamma (PPARy), which is a receptor that acts as a tumor suppressor in colorectal cancer. Itaconate may be a link between leptin, obesity, and colorectal cancer development [40]. More research is required to further clarify this complex relationship.

The alterations of the gut microbiome in individuals with obesity may also be a contributory factor to CRC development. Multiple studies have been published regarding the differences in gut microbiota profile in patients with CRC and the possible role for a metabolic and microbial marker [41–44]. It is currently unclear if the differences in the gut microbiome between patients with and without CRC are part of the cause or effect. However, the gut microbiome of patients with obesity also differs to their non-obese counterparts, potentially augmenting their CRC risk. *Peptostreptococcus stomatis* leading to dysregulation of fatty acids and phospholipids have been linked to colorectal cancer patients with obesity as the cross-feeding behavior of species *P. stomatis* promotes tumorigenesis [45]. Decreased bacterial diversity leading to reduction in short chain fatty acid (SCFA) producing bacteria, such as *Roseburia* and *Faecalibacterium prauznitzii,* in the colon has been demonstrated in patients with type 2 diabetes and in obesity. Butyrate, a SCFA, is important in strengthening the intestinal barrier of the mucosa and stimulating anti-inflammatory cytokine production [39].

There are many factors that contribute to the pro-inflammatory state induced by obesity and specifically, VAT. When there is excess adipose tissue, it will undergo “adipose tissue remodeling” to increase the size of mature adipocytes [11]. During this process, inflammatory cytokines such as tumor necrosis factor- α (TNF- α), interleukin-1 (IL-1), IL-6, IL-12, and IL-23 are secreted. Furthermore, the expansion of mature adipocytes leads to increased hypoxia and oxidative stress due to insufficient vascular oxygenation [11]. IL-6, which can increase C-reactive protein, stimulates fibroblasts to produce vascular endothelial growth factor (VEGF) promoting neovascularization and tumorigenesis in sporadic CRC [46]. IL-6 has also been linked to advanced stage of CRC and decreased survival. Experimental studies demonstrated that IL-6’s effects are mediated through signaling through Janus kinases (JAKs) signal transducer and activator of transcription 3 (STAT3) [47]. TNF- α influences the Wnt/β-catenin pathway and PI3K/AKT pathway which have been found to be involved in the development of cancers, including CRC [46, 47]. The impact of obesity’s pro-inflammatory effects in CRC development and progression are multifactorial and present an opportunity for targeted treatment.

## Weight Loss in Augmenting Colorectal Cancer Risk and Outcomes

Given the well-established relationship between obesity and CRC risk, the question arises of whether weight loss can reduce the risk of CRC in individuals with obesity. Bariatric surgery has been demonstrated to result in sustained weight loss in addition to remission of obesity-associated medical problems (i.e. type 2 diabetes, hypertension) [48]. Bariatric surgery has been shown to significantly reduce the risk of CRC development in patients with obesity [49–51]. Pararas et al. demonstrated a 44% risk reduction (95% CI 0.4–0.8) after bariatric surgery, which appears to be consistent when compared to other recent meta-analyses. These authors performed an additional subgroup analysis comparing sleeve gastrectomy with gastric bypass and found that sleeve gastrectomy was significantly associated with fewer cases of CRC (45% reduction, 95% CI 0.36–0.83), while no significant effect was found when assessing gastric bypass [52].

Chieri et al. investigated whether bariatric surgery also reduced CRC risk in individuals with *morbid* obesity. This meta-analysis found that individuals who underwent bariatric surgery had a 54% risk reduction for CRC (95% CI 0.28–0.75). A 46% risk reduction (95% CI 0.37–0.79) in *women* with a history of bariatric surgery was also observed, but the results were non-significant in men. It is important to note there was marked heterogeneity in time of follow-up amongst the studies included in this meta-analysis, ranging from 3 years to over 20 years. To mitigate this bias, the authors also assessed hazard ratios which demonstrated a 19% reduction in CRC risk for patients with obesity who had a history of bariatric surgery [48]. In contrast to the findings of Pararas et al., Chieri et al. demonstrated no significant difference when comparing sleeve gastrectomy and gastric bypass [48]. Regardless of type of bariatric surgery, the time it takes for weight reduction to translate into CRC risk reduction is not well established. However, some studies have demonstrated a protective effect as early as five years after bariatric surgery [53]. Overall, studies are limited by the inherent latency of CRC and further prospective studies would be beneficial in characterizing this risk.

Bariatric surgery results in remission of metabolic disorders – T2D, MASLD, and hyperlipidemia—which are independent risk factors for CRC development [48, 54–57]. The underlying mechanisms affect CRC risk after gastric bypass in particular are not clearly elucidated. Gastric bypass has been demonstrated to promote an anti-inflammatory effect due to decreased macrophage infiltration [57]. However, individuals after gastric bypass also have demonstrated changes in their gut microbiome possibly leading to increased exposure of colonic mucosa to bile acids resulting in growth of pro-inflammatory bacteria and a decrease in butyrate-producing bacteria [48]. Nevertheless, these findings remain largely hypothetical and may not be clinically significant as multiple observational meta-analyses have demonstrated a protective effect of bariatric surgery, regardless of subtype (sleeve gastrectomy versus gastric bypass) [49–51].

Medical therapy, specifically glucagon-like peptide-1 (GLP-1) agonists, are becoming increasingly popular for glucose control and weight loss. For this reason, research into its effect on CRC risk has become an area of interest. A meta-analysis by Figlioli et al. demonstrated no significant impact of GLP-1 agonists on CRC risk. It is important to note that the longest follow-up period assessed in the subgroup analyses was ≥ 5 years of follow-up, and therefore, may not have allowed for an appropriate follow-up period in the setting of the latent nature of CRC [58]. A recent Mendelian randomization demonstrated an *increased* risk of CRC (RR 1.12, 95% CI 1.07–1.18) with GLP-1 agonist usage. The authors acknowledged that most of the studies had shorter follow up periods, with only a small proportion of the included studies having a follow-up greater than two years [59]. Future studies including randomized control trials with long-term follow up would be beneficial.

Independent from weight loss surgeries and medications, prehabilitation programs have also been shown to decrease the severity and incidence of post-operative medical complications after colorectal cancer surgery in individuals with obesity [60]. Molenaar et al. performed a randomized control trial that found that a 4-week standardized prehabilitation program in addition to standard postoperative care led to decrease in cardiac and respiratory complications, although surgical outcomes and patient postoperative walking time was equivalent. The prehabilitation program included a multidisciplinary approach with a standardized 4-week in-hospital high-intensity exercise regimen three times per week supervised by therapists, regular dietary assessments and interventions by a dietician, as well as psychological support via providing anxiety-coping mechanisms by psychology trained personnel. An ideal time to implement such a regimen would be during the neoadjuvant treatment period, as it would also prevent delay in surgery. There is a paucity of data assessing the relationship between pre-operative weight loss in patients with obesity from bariatric surgery and GLP-1 agonists and intra-operative/post-operative complications and mortality.

## Obesity and Treatment of Colorectal Cancer

Obesity increases risk of CRC development while also affecting prognosis and outcomes. A meta-analysis by Doleman et al. found that patients with obesity had an increased risk of all-cause mortality (RR 1.14, 95% CI 1.07–1.21), cancer-specific mortality (RR 1.50, 95% CI 1.20–1.87), recurrence (RR 1.07, 95% CI 1.02–1.13), and worse disease-free survival (DFS [RR 1.07; 95% CI 1.13–1.43]) compared to patients with normal weight. Therefore, it is crucial to evaluate the impact of obesity on medical and surgical treatments in patients with CRC.

### Chemotherapy and Immunotherapy

Many new studies have been recently published evaluating the relationship between obesity and response to medical treatment, such as chemotherapy or immunotherapy, in CRC patients. In patients who have a grade 1 to 2 tumor undergoing hepatectomy after neoadjuvant chemotherapy, a longer progression-free survival (PFS) has been demonstrated in patients < 24 kg/m^2^. These results were not stratified to assess the difference in PFS in patients with higher BMI (i.e. overweight versus obese) [61]. In contrast, there are recent studies that suggest obesity may allow certain types of chemotherapy and immunotherapy to be more efficacious in colorectal cancer. Cybulska-Stopa et al. demonstrated that patients with metastatic colorectal cancer and a higher BMI who were treated with bevacizumab in *second line* treatment had an improved prognosis in longer PFS. However, this study was limited by lack of KRAS data for over 41% of the study participants (which was strongly associated with overall survival [OS]) and its retrospective nature [62].

Cortellini et al. showed that tumor objective response rate (ORR) was higher in overweight/obese patients compared to non-overweight patients for those treated with anti-PD-1/PD-L1 inhibitors. PFS and OS were longer for overweight/obese patients who were treated with anti-PD-1/PD-L1 inhibitors [63]. CD4 + T cell dysfunction leads to increased PD-1 expression in obese patients [64]. This may be the underlying mechanism for why these patients showed an improved response to anti-PD-1/PD-L1 inhibitors. CRC patients with increased BMI should also have weight-based chemotherapy and immunotherapy doses [65]. Providers should remain vigilant in assessing for therapy-induced toxicities and treat accordingly.

### Colorectal Cancer Surgery in Patients with Obesity

Minimally invasive techniques for CRC surgery have been utilized for over 25 years [66]. There is no difference in oncologic outcomes – including lymph node yield, local or distant recurrence, 5-year DFS and OS – when using minimally invasive techniques for CRC surgery in individuals with obesity compared to those without [67, 68]. Furthermore, minimally invasive techniques have been demonstrated favorable outcomes including a lower mortality rate, lower complication rate, lower ostomy rate, shorter LOS, higher routine discharge rate, and lower overall cost when compared to open techniques [68]. Robotic colorectal surgery was first performed successfully in 2001 and has since become a widely adopted approach in colorectal surgery [66]. Robotic surgery has been demonstrated to be safe and have similar short-term outcomes when compared to laparoscopic surgeries, however, use can be limited by availability, surgeon experience, and cost [66, 67].

For rectal cancer in particular, the standard of care is total mesorectal excision (TME). Mid and low rectal cancers can be especially challenging laparoscopically due to narrow pelvic spaces, especially in individuals with obesity which can make it difficult to achieve a high quality TME. Nevertheless, a randomized clinical trial by Jiang et al. demonstrated comparable pathologic outcomes including comparable TME, negative circumferential margins, distal resection margins, and number of lymph nodes retrieved when laparoscopy was compared to open techniques [69]. Colorectal surgery can be technically challenging in patients with obesity [70]. Robotic surgery has become a popular surgical modality in patients with obesity to help mitigate the technical challenges by enhancing ergonomics, allowing for increased degrees of freedom in the narrow space like the pelvis, and improved visualization.

It is well established that obesity is associated with higher risk of post-operative surgical complications [71–73]. With BMI assessed as a continuous model, increased risks of postoperative complications such as prolonged ileus, postoperative fluid collections, wound infection, and anastomotic leak have all been demonstrated [13]. Post-operatively, patients with increased BMI have been observed to have increased time to flatus and higher rates of reoperation [12]. In rectal cancer, obesity is an independent risk factor for anastomotic leakage, possibly due to increased technically difficulty, factors contributing to poor postoperative healing including metabolic co-morbidities that are often associated with obesity like T2D and peripheral arterial disease [70]. The increased rates of surgical site infections (SSIs) in patients with obesity may be explained by a different underlying causes. Obesity is linked to adipocyte remodeling leading to expansion of adipocytes and decreased oxygen perfusion, decreased lymphocyte function, promotion of a pro-inflammatory state, and possibly decreased therapeutic concentration doses in prophylactic antibiotics [13, 48]. There are techniques that are proposed to help decrease the risk of SSIs in this population. Negative pressure wound therapy (NPWT) dressings on the surgical site have become increasingly popular to aid wound healing – theoretically due to increased wound contracture and granulation tissue by increasing capillary blood flow, endothelial proliferation, and angiogenesis [74, 75]. A single institutional study by Carrano et al. demonstrated no difference in SSI incidence after stoma-reversal when using NPWT, but higher aesthetic satisfactions in uninfected wounds [76]. A meta-analysis by Sahebally et al., found that NPWT application reduced SSI rates in colorectal surgery (pooled OR 0.16, 95% CI 0.07–036) with no difference in seroma (pooled OR 0.38, 95% CI 0.12–1.23) or wound dehiscence (pooled OR 2.03, 95% CI 0.61–6.78) in laparotomy wounds in general. Larger randomized control trials and prospective are needed to further assess if there is a benefit in patients with obesity Table 2.

**Table 2:** Summary of studies assessing surgical outcomes in CRC patients with obesity from 2019–2024

Study	Type of Study	Sample Size	Population	Outcome	Key Findings
Cullinane et al. (2023) [71]	Meta-analysis	1,866,326	Patients with obesity (BMI ≥ 30 kg/m^2^)	30-day morbidity and mortality after general surgery procedures	Obesity (*when all obesity classes are combined*) was associated with an increased risk of 30-day postoperative morbidity (OR 1.11, 95% CI 1.04–1.11) including a higher rate of SSI (OR 1.40, 95% CI 1.24–1.59) No significant difference in in-hospital mortality between patients with and without obesity (OR 1.15, 95% CI 0.79–1.67)
Xu et al. (2020) [73]	Meta-Analysis	60,229	Patients who underwent colorectal cancer surgery	Surgical site infection	Patients with obesity (BMI ≥ 30 kg/m^2^) had an increased risk of SSI (OR 1.59, 95% CI 1.40–1.81)
Nugent et al. (2021) [72]	Meta-Analysis	32,953	Colorectal cancer patients with obesity	Anastomotic Leak Rates	Anastomotic leak rate was statistically increased in those with obesity in both Western and Asian cohorts However, clinical significance was only observed in the sub-analysis for *rectal* anastomosis in both Western (OR 1.57, 95% CI 1.01–2.44) and Asian (OR 1.58, 95% CI 1.07–2.32) cohorts
Wee et al. (2019) [78]	Meta-Regression Analysis	262	Colorectal cancer patients with obesity who undergo robotic colorectal surgery	Post-operative outcomes within 30 days of surgery	Robotic surgery provided earlier recovery with shorter LOS (MD -2.55 days, 95% CI -3.13 to -1.97 days) and lower risk of re-admission (RR 0.42, 95% CI 0.19–0.92)
Wang et al. (2023) [67]	Systemic Review and Meta-Analysis	663	Colorectal cancer patients with obesity who undergo robotic colorectal surgery	Length of stay (LOS), surgical site infection (SSI) rate, complications, anastomotic leak and oncological outcomes	In comparison to open or laparoscopic surgery, robotic surgery demonstrated no significant difference in operative time, conversion to open procedure, LOS, lymph node yield, anastomotic leak, and postoperative ileus There is a trend present of increased complications in individuals with obesity compared to those without obesity (32.3% vs. 26.8% for complications and 14.2% vs. 9.9% for SSIs) Robotic surgery for colorectal cancer is safe in patients with obesity

*OR* odds ratio, *CI* confidence interval, *LOS* length of stay, *SSI* surgical site infections

## Conclusion

Obesity is an independent risk factor for the development of CRC. It has also been shown to lead to worse oncologic and post-operative outcomes including SSIs and mortality. At present, there are no established guidelines on whether colorectal patients with obesity should undergo different screening, treatment, or follow-up care. While bariatric surgery has been shown to decrease risk of CRC, newer treatments like GLP-1 agonists have mixed results. Prehabilitation programs are the only methods of pre-operative weight loss that have been proven to decrease post-operative morbidity in this population. Current gaps in etiologic knowledge include the exact understanding of the underlying biological mechanisms that link obesity and CRC, role of adipose tissue in the TME, heterogeneity in CRC risk amongst different racial/ethnic/demographic populations, timing and duration of obesity, and the impact of timing of weight loss on CRC risk. Whether obesity should be considered as a metric to offering earlier screening and whether surgical and medical weight loss techniques can contribute to improving post-operative morbidity are among current therapeutic knowledge gaps. More long-term studies are needed to assess the effects of these new and developing treatments on colorectal cancer risk and outcomes.

## Key References

- Bardou, M, Rouland A, Martel M, Loffroy R, Barkun AN, Chapelle N. Review article: obesity and colorectal cancer. Aliment Pharmacol Ther. 2022;56(3):407-18
  - A great summary of the up-to-date literature and practices related to obesity and colorectal up to 2022, building upon the author’s previous work from 2013.
- Chaplin, A, Rodriguez RM, Segura-Sampedro JJ, Ochogavia-Segui A, Romaguera D, Barcelo-Coblijn G. Insights behind the Relationship between Colorectal Cancer and Obesity: Is Visceral Adipose Tissue the Missing Link? Int J Mol Sci. 2022;23(21).
  - An excellent, key review in reviewing the current understanding on the epidemiology of obesity and colorectal cancer risk.
- Chierici A, Amoretti P, Drai C, De Fatico S, Barriere J, Schiavo L, et al. Does Bariatric Surgery Reduce the Risk of Colorectal Cancer in Individuals with Morbid Obesity? A Systematic Review and Meta-Analysis. Nutrients. 2023;15(2).
  - An excellent meta-analysis that delves into the impact of bariatric surgery and how it reduces the risk of colorectal cancer. This study also incorporates a separate metric (hazardratio) that evaluates the impact of history of bariatric surgery in colorectal cancer risk regardless of time to follow up which is unique amongst the recent meta-analyses.

## Data Availability

No datasets were generated or analysed during the current study.
